# Effect of mass sports activity on prosocial behavior: A sequential mediation model of flow trait and subjective wellbeing

**DOI:** 10.3389/fpubh.2022.960870

**Published:** 2022-08-01

**Authors:** Xiyan Duan, Xiaohua Wang, Xiaogang Li, Shichen Li, Yiping Zhong, Te Bu

**Affiliations:** ^1^College of Physical Education, Hunan Normal University, Changsha, China; ^2^School of Physical Education and Health, Wenzhou University, Wenzhou, China; ^3^Cognition and Human Behavior Key Laboratory of Hunan Province, Department of Psychology, School of Education Science, Hunan Normal University, Changsha, China

**Keywords:** health policy, mental health, physical activity, dispositional flow, empathy, leisure

## Abstract

**Objectives:**

Participation in mass sports is one of the most efficient strategies for people to attain physical and mental health in China. Prosocial behavior has a positive effect on social development. This study developed a conceptual model with mass sports activity as the independent variable, prosocial behavior as the dependent variable, and flow trait and subjective wellbeing as the mediating variables.

**Methods:**

Participants (*N* = 351) completed an online survey. Mass sports activity, flow trait, subjective wellbeing, and prosocial behavior were measured using the physical activity rank scale-3 (PARS-3), short dispositional flow scale (SDFS), index of wellbeing (IWB), and prosocial tendencies measure (PTM), respectively. Descriptive statistics compared differences between sports population (PARS-3, ≥ 36) and non-sports population (PARS-3, <36). Mediation effect was analyzed using the PROCESS (Template, Model 6).

**Results:**

Sports population scored significantly higher (all *P* ≤ 0.05) on SDFS, IWB, and PTM than non-sports population. Participation in mass sports stimulated flow trait and thus improved prosocial behavior, with a mediation effect value of 0.061 (95% *CI*, 0.028–0.104), which accounted for 30.18% of the total effect. Participation in mass sports enhanced subjective wellbeing and thus improved prosocial behavior, with a mediation effect value of 0.044 (95% *CI*, 0.007–0.090), which accounted for 21.96% of the total effect. Flow trait and subjective wellbeing mediated the relationship between mass sports activity and prosocial behavior in a sequential manner, with a mediation effect value of 0.059 (95% *CI*, 0.035–0.090), which accounted for 29.23% of the total effect.

**Conclusion:**

The preliminary results of the mediation model validated the hypothesized sequential links between mass sports activity, flow trait, subjective wellbeing, and prosocial behavior. Greater participation in mass sports increases the likelihood of prosocial behavior.

## Introduction

Prosocial behavior is a crucial aspect of an individual's socialization and refers to any behavior that matches social norms and is beneficial to individuals, groups, or society (1). Prosocial behavior typically demonstrates admirable attributes such as modesty, assisting, cooperation, and sharing in interpersonal interactions (2), which is a positive habit that society promotes for development. Social learning theory argues that individuals can develop prosocial behavior through witnessing the behaviors and outcomes of others during physical activity, learning, and reinforcing the behavioral outcomes (3). Individuals establish good interpersonal relationships with their peers through learning the rules of sports and imitating the role of sports (4). In physical activity, individuals also learn to deal with the relationship between competition and cooperation in a harmonious manner, and they acquire prosocial qualities such as humility, respect, solidarity, and helping others (5). Personal behavior, according to the theory of group dynamics, is the outcome of the interaction between the intrinsic demands of the individual and the external forces of the environment (6). On the basis of this notion, empirical studies have showed that physical activity facilitates the development of prosocial behavior in adolescents (7) and adults (8). For example, Moeijes and colleagues investigated longitudinal relationships between 10 and 12-year-olds' sports participation and prosocial behavior (9). Membership in a sports club and moderate or frequent sports participation were found to be longitudinally correlated with improved prosocial behavior. Although previous research have demonstrated the positive relationship between small group-based exercise, competitive sports, and prosocial behavior, the effect and its underlying mechanisms by which leisure physical activity influences prosocial behavior remain unexplained.

The concept of fitness for all has been integral to the development of the People's Republic of China since its inception. In 2016, the Central Committee of the Chinese Communist Party and the State Council issued the Outline of the “Healthy China 2030” Plan (10), which further encourages the Chinese public to participate actively in sports and scientific fitness. Mass sports activity is a type of popular sports activity (e.g., walking, Tai Chi, badminton, and marathon) that strives to increase physical fitness, health, leisure activities, and social feelings for all members of Chinese society (11). Zhu and Han researched the effects of different sports participation on prosocial behavior and showed that leisure sports participants scored higher than competitive sports participants on measures of prosocial behavior (12). Specifically, leadership, social facilitation, and group cohesion were found as three specific prosocial traits where leisure sports participants outperformed competitive sports participants. In light of this study, mass sports activity may not only be useful for promoting population physical health, but also for fostering social solidarity, which is absent from the current research context in China.

Flow theory is a field of research within positive psychology. Flow is the best condition of experience in which an individual is concentrating deeply on an activity, completely immersed, totally engaged, highly enjoyable, and experiencing favorable feelings during the action (13). State and trait of flow are distinct components of flow experience (14). Flow trait reflects the frequency with which individuals experience flow throughout an event, whereas flow state captures the sensation of flow during an event. Flow in sports and its applications to prosocial behavior are a relatively young area of research. A few studies on elite athletes suggest that physical activity may positively influence the flow experience (15, 16). Li and Zhang (17) found that easy-to-learn tennis instruction can motivate novices to learn and increase the likelihood of students experiencing the state of flow. Therefore, simple and popular mass sports activity may be beneficial at attracting the interest of individuals and stimulating their flow experience. Meanwhile, flow experience has been recommended to be more conducive to promoting prosocial behavior (18). Traditionally, research on flow in the context of sports has focused on its effects on the performance outcomes of elite athletes, whereas its social psychological benefits associated with leisure physical activity have received less attention. There is a need for rigorous study to determine whether the flow experience induced by mass sports activity could be a precursor to prosocial behavior.

Subjective wellbeing is a holistic evaluation of an individual's quality of life and is a comprehensive psychological indicator of personal and social quality (19). In healthy persons, there is a beneficial correlation between physical activity and subjective wellbeing (20). Chatzisarantis and Hagger found that leisure sports participants experienced greater psychological wellbeing than competitive sports participants (21). In recent years, the central and municipal governments of China have increased expenditure on sports infrastructure in an effort to foster mass sports activity (22). As a result, the nationwide urban fitness trails has expanded from 823,500 in 2019 (23) to 929,300 in 2021 (24). The provision of urban sports facilities at the grassroots level could promote not only leisure sports activity and physical health (25), but also social engagement (26). A case study conducted in Zhuhai City, China showed that community sports parks have a positive influence on the subjective wellbeing of community members (27). Participation in mass sports could therefore improve mental health in contemporary Chinese society. Meanwhile, subjective wellbeing reinforces prosocial behavior (28). The relationship between subjective wellbeing and prosocial behavior among Chinese population has been proven to be both positive and bidirectional (29, 30). Despite this, the influence of leisure physical activity on positive psychology is an understudied topic. Particularly in light of the growing acknowledgment of antisocial behavior as a serious public health concern, positive psychology-based research on mass sports activity is warranted.

Therefore, this study analyzed the mechanism underlying the influence of mass sports activity on prosocial behavior and developed a theoretical foundation for increasing mental health through physical health. It was hypothesized that mass sports activity has a positive direct relationship with prosocial behavior, and that flow trait and subjective wellbeing mediate the relationship between mass sports activity and prosocial behavior. The multiple mediator model covers parallel mediator model and serial mediator model. In a parallel mediation model, the mediating variables have no effect on one another. The serial mediation model indicates that the mediating variables exert mutually influential effects. Wu et al. (31) suggested that the greater the degree of flow experience, the greater the likelihood that it can induce subjective wellbeing, and that the two are linked. Further, it was theorized that the flow experience induced by mass sports activity could enhance the subjective wellbeing of individuals, hence sequentially mediating and encouraging prosocial behavior.

## Methods

### Participants

The research was approved by the Ethics Committee of Hunan Normal University. All study participants or their legal guardians, if they were younger than 18 years old, provided informed consent (signed online during the survey). Using G^*^Power (version 3.1), the multiple linear regression *R*^2^ one-sample procedure was chosen to predict the sample size with significance level of 0.05 and a statistical test power of 0.95. The *priori* analysis estimated a sample size of at least 107 to detect a predictive effect of mass sports activity and prosocial behavior (*f*^2^ = 0.15). Data were collected using an online survey platform (Credamo, China) between March and May 2021. Credamo delivered surveys at random to personnel in all regions of China, and 367 surveys were collected in total. Excluding 16 surveys whose testing questions were unsatisfactory or too brief, a total of 351 valid surveys were retrieved, yielding an effective response rate of 95.64%. A total of 172 males and 179 females participated in this survey. There were 20 participants under the age of 18, 231 participants between the ages of 19 and 30, 95 participants between the ages of 31 and 45, three participants between the ages of 46 and 59, and two participants over the age of 60. This sample includes 102 students, 244 full-time workers, and five individuals who were unemployed or retired.

### Instruments

Respondents' participation in mass sports over the last month was measured using the physical activity rank scale-3 (PARS-3) (32). PARS-3 ranked the intensity of physical activity (“What level of physical exertion do you engage in?” score range: 1–5 points), duration of physical activity (“How many minutes at a time do you engage in the physical activity described above?” score range: 1–5 points), and frequency of physical activity (“How frequently do you participate in the aforementioned physical activities?” score range: 0–4 points). The total score (PARS-3 = intensity × time × frequency) ranges from 0 to 100. China's National Fitness Program (33) defines the sports population as individuals who exercise moderately at least three times per week for at least 30 min per session. In this study, individuals with scores of 36 or more were considered part of the sports population, while those with scores of <36 were considered part of the non-sports population. The questionnaire has proven good test-retest reliability (*r* = 0.82) (32) and has been widely utilized in China to assess physical activity.

Flow trait was measured using the short dispositional flow scale (SDFS; 5-point Likert scale: 1, never experienced a flow; 5, always experienced a flow) (34). SDFS measured nine dimensions of flow: challenge—skill balance, merging of action and awareness, clear goals, unambiguous feedback, concentration on the task at hand, sense of control, loss of self-consciousness, transformation of time, and autotelic experience. All dimensions were added together to determine the total score. Higher scores indicate an individual's perception of flow trait in physical activity. Cronbach alpha of this study population was 0.76.

Subjective wellbeing was measured using the index of wellbeing (IWB; 7-point Likert scale: 1, strongly unsatisfied; 7, strongly satisfied) (35). IWB is comprised of index of general affect (containing of eight items with a score weight of 1) and index of life satisfaction (containing of one item with a score weight of 1.1). The total score (IWB = mean of eight items in the index of general affect ×1 + one item in the index of life satisfaction ×1.1) ranges from 2.1 to 14.7. The higher the score, the happier the respondent. Cronbach alpha of this study population was 0.92.

Prosocial behavior was measured using the prosocial tendencies measure (PTM; 5-point Likert scale: 1, does not describe me at all; 5, describes me greatly) (36). PTM consists of a total of 23 items, which are categorized into six dimensions: altruism, dire, compliant, emotional, public, and anonymous. All dimensions were added together to determine the total score. Higher scores imply that the respondent's prosocial tendencies are more evident. Cronbach alpha of this study population was 0.86.

### Statistics

Data were analyzed using the IBM SPSS Statistics (version 25.0) and Hayes' PROCESS (version 4.1). Statistical significant level was set at *P* < 0.05. First, a two-tailed Welch's *t*-test was conducted to determine if the sports and non-sports populations differed significantly in the PARS-3, SDFS, IWB, and PTM. Then, the correlations between mass sports activity, flow trait, subjective wellbeing, and prosocial behavior were computed, and a linear regression was used to determine if mass sports activity had a significant influence on prosocial behavior. Finally, the PROCESS (Template, Model 6) was utilized to examine whether trait flow and subjective wellbeing served as sequential mediators between mass sports activity and prosocial behavior. The study utilized a bias-corrected non-parametric percentile bootstrap method appropriate for testing the significance level of the mediation effect, with 5,000 random resampling from the total sample size (*N* = 351), and the mediation effect was significant if the 95% confidence interval did not cross zero (37).

## Results

### Common method bias

Survey research often introduces data bias due to the characteristics of the survey items or the consistency of the data sources. In order to increase the truthfulness of the survey, it was constructed with lie detector and individuals were instructed to complete it anonymously. The Credamo was used to collect surveys nationwide in order to avoid consistency in questionnaire origin. Harman's single-factor test was employed to confirm the common method bias. The unrotated factor analysis examined a total of 10 common factors with eigenvalues were >1, the first of which explained 24.81% of the variance, which is below the critical threshold of 40%. This suggests that the common method bias did not cause significant issue in this study.

### Descriptive statistics

Table 1 shows the descriptive statistics of variables. The SDFS, IWB, and PTM scores of sports population were significantly higher than those of non-sports population. On the PARS-3, male scored significantly higher than female. Likewise, male's SDFS, IWB, and PTM scores were significantly higher than female's. These findings indicate that participation in mass sports could effectively influence the extent to which trait flow, subjective wellbeing, and prosocial behavior tendencies are experienced.

**Table 1:** Means ± standard deviations of variables.

	**Sports population**			**Sex**		
	**No (*n* = 287)**	**Yes (*n* = 64)**	***P***	**Female (*n* = 179)**	**Male (*n* = 172)**	***P***
Physical activity rank scale-3	15.72 ± 8.45	41.61 ± 9.38	<0.001	18.66 ± 12.47	22.30 ± 13.72	0.01
Short dispositional flow scale	3.88 ± 0.48	4.09 ± 0.45	0.001	3.83 ± 0.51	4.01 ± 0.44	0.001
Index of wellbeing	11.69 ± 2.00	12.13 ± 1.54	0.05	11.40 ± 2.12	12.15 ± 1.62	<0.001
Prosocial tendencies measure	83.63 ± 10.84	87.94 ± 10.50	0.004	83.08± 10.65	85.81 ± 11.00	0.019

### Correlation analysis

Table 2 summarizes correlations among factors. The correlation analysis between the variables revealed significant positive relationships between mass sports activity, trait flow, subjective wellbeing, and prosocial behavior (all *P* < 0.001). The regression coefficients between the variables were determined using a linear regression model, and the results are presented in Table 3. The variance inflation factor values of all predictor variables in this study are below five, hence there is no multicollinearity issue (38). The result of simple linear regression supports hypothesis one, demonstrating that mass sport activity significantly predicted prosocial behavior, β = 0.243, *t*_(349)_ = 4.687, *P* < 0.001.

**Table 2:** Matrix of Pearson correlation coefficient for variables.

	**1**	**2**	**3**	**4**
Mass sports activity	1			
Flow trait	0.339^***^	1		
Subjective wellbeing	0.307^***^	0.560^***^	1	
Prosocial behavior	0.243^***^	0.460^***^	0.542^***^	1

*** *Denotes P < 0.001*.

**Table 3:** Results of regression analysis.

**Outcome variable**	**Independent variables**	***R***	***R* ^2^**	***F***	**β**	***t***	***P***	***df***	**VIF**
Prosocial behavior	Mass sports activity	0.243	0.059	21.971	0.243	4.69	<0.001	349	1.00
Flow trait	Mass sports activity	0.339	0.115	45.441	0.339	6.74	<0.001	349	1.00
Subjective wellbeing	Mass sports activity	0.574	0.329	85.414	0.131	2.82	0.005	348	1.13
	Flow trait	-	-	-	0.516	11.05	<0.001	348	1.13
Prosocial behavior	Mass sports activity	0.575	0.331	57.176	0.045	0.96	0.338	347	1.16
	Flow trait	-	-	-	0.216	3.99	<0.001	347	1.53
	Subjective wellbeing	-	-	-	0.407	7.58	<0.001	347	1.49

*VIF, variance inflation factor*.

### Two-mediator sequential model

**Figure 1:**
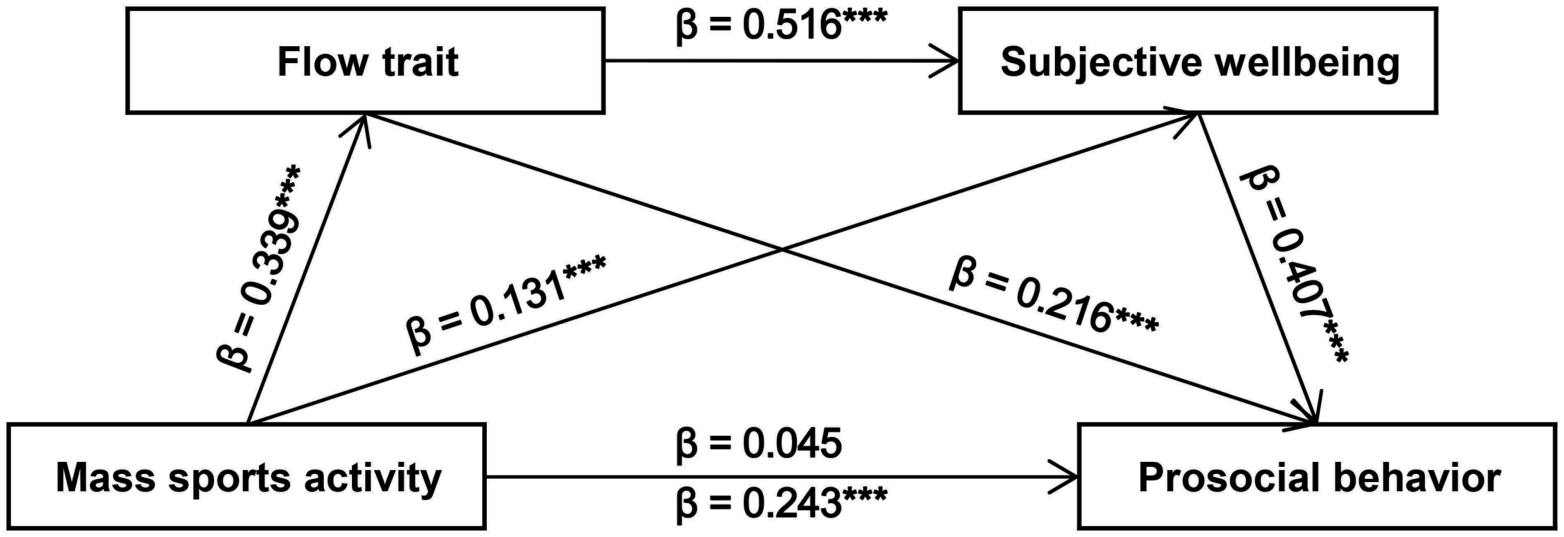
Conceptual diagram of double mediation using the PROCESS (Template, Model 6). Beta values above the solid line represent indirect effects, while the beta value below the solid line represent the total effect. ***Denotes *P* < 0.001.

Table 4 presents the mediation effects based on the bootstrap method. The 95% confidence intervals of all three indirect paths do not contain zero, indicating that all three paths have significant mediation effects. Furthermore, the significance of mass sports activity on prosocial behavior disappeared (c' = 0.037, *P* = 0.338) after the inclusion of two mediators, indicating that flow trait and subjective wellbeing had a fully mediation effect in this study. Therefore, the validity of hypotheses two, three, and four is supported by these findings. Figure 1 depicts the two-mediator sequential model.

**Table 4:** Results of sequential mediation analysis.

**Indirect effect path**	**Effect**	**Boot *SE***	**Boot 95% *CI***	**Ratio of indirect to total effect of *X* on *Y***
Ind1: *X*→*M*_1_→*Y*	0.061	0.019	[0.028, 0.101]	30.18%
Ind2: *X*→*M*_2_→*Y*	0.044	0.021	[0.007, 0.089]	21.96%
Ind3: *X*→*M*_1_→*M*_2_→*Y*	0.059	0.014	[0.035, 0.089]	29.23%
Indirect effects of *X* on *Y*	0.163	0.031	[0.108, 0.230]	81.37%
Total effect of *X* on *Y*	0.201	0.043	[0.117, 0.285]	-

*X, mass sports activity; M_1_, flow trait; M_2_, subjective well-being; Y, prosocial behavior*.

## Discussion

In this study, we showed that participation in mass sports can promote prosocial behavior. Based on the subgroup analysis, it was also identified that the sports population scored significantly higher on flow trait, subjective wellbeing, and prosocial behavior, highlighting the value of leisure physical activity. The current study is the first to demonstrate positive psychological outcomes in addition to physical health benefits associated with mass sports activity, so providing the theoretical underpinning to support the fitness for all policy of the Chinese government.

Sports participation throughout the lifespan is regarded to enhance moral character and the capacity to work collaboratively toward a common objective (39–41). Individual sports participation is governed by the sport's laws and ethics, which are gradually reinforced as the number of sports activity increases (42). This is congruent with the social norms theory (43), which argues that norms can be internalized into an individual's consciousness (44) and can be adhered to even in the absence of external rewards (45). Thus, individuals with active inclusion in mass sports exhibit more pronounced prosocial behavior. Furthermore, mass sports are sports activities conducted in public, and people can be influenced by the watching eyes effect (46) when participating in sports, thereby creating an implicit social pressure to be supervised, and are more likely to develop prosocial behavior as a result of this supervision mechanism (47).

In the present study, flow trait and subjective wellbeing independently mediated the relationship between mass sports activity and prosocial behavior, respectively. According to the flow theory, this is mostly due to the fact that when people engage in easy-to-learn mass sports activity, they concentrate on the task at hand while experiencing a sense of joy and fulfillment (48). During the flow process, the individual's sense of control rises (49), and he or she is also more inclined to exhibit prosocial behaviors such as helping, cooperation, and humility (50). Furthermore, numerous research has demonstrated that regular physical activity, such as walking (51), could bring about array of benefits on an individual's physical and mental health, resulting in a relaxed and content state of mind and body. Meta-analysis of leisure sports participation and subjective wellbeing suggested that the leisure domain is an essential target for boosting subjective wellbeing (52), which has been demonstrated in the Chinese (53) and European (54) population. Mass sports activity is a form of leisure physical activity in which individuals of all ages can participate at any time. Physical activity improves individuals' subjective wellbeing, and in turn, when people are in a state of happiness, they are more inclined to engage in prosocial behavior (55).

Through the sequential mediation effect of flow trait and subjective wellbeing, the present study shows that mass sports activity can predict prosocial behavior. Our conclusion is consistent with the literature that flow is a strong predictor of subjective wellbeing (31). The sense of flow that people develop while exercise results in a heightened sense of wellbeing, which encourages prosocial behavior. This effect can also be explained by the theory of empathy training (56). Empathy is the capacity of the observer to feel and comprehend the feelings of the observed, as well as the mental process by which humans recognize and experience the emotions and feelings of others. Sport education (57) and regular participation in organized physical activity (58) can enhance an individual's personal and social responsibility, and a higher level of physical activity correlates with a greater capacity for empathy (59). Given the relationship between heightened flow experience and empathy (60), as well as the link between positive empathy, subjective wellbeing, and prosocial behavior (61), mass sports activity contributes to more prosocial behavior.

This study's findings have significant practical implications. First, our data revealed a significant gender difference in physical activity, which resulted in a significant decrease in females' flow trait, subjective wellbeing, and prosocial behavior. Insufficient physical activity among females is prevalent not only in China, but worldwide (62). In response to this global trend, governments should implement more effective health campaigns to encourage female physical activity. Not only could active sports participation improve physical health, but it could also have a positive influence on mental health, as demonstrated in the present study. This recommendation also applies to disadvantaged populations, such as those with physical disabilities or who live in economically underdeveloped areas with fewer public fitness facilities. Second, the sequential mediation effect of flow trait and subjective wellbeing suggests that individuals may choose appropriate exercise form (e.g., pacing sports) (16), extend the exercise duration, and increase the exercise intensity (63) when performing mass sports activity in order to achieve a deep state of flow in sports, which can increase the level of subjective wellbeing after exercise and improve the prosocial behavior of individuals. Third, these findings have implications for physical education classes in schools and team-building training in businesses. By organizing extracurricular and leisure physical activity, schools and businesses can increase the level of prosocial behavior among students and employees, hence enhancing the efficacy of the class and business (64).

The Chinese government has promoted the construction of “Healthy China” and developed an innovative public health model with Chinese characteristics to intervene in life and guide social behavior through sports, which not only has a positive impact on China's social progress and development, but also serves as an excellent example for the international community. On the basis of the Chinese experience, the promotion of an active lifestyle and social cohesion can be more effectively done through a series of societal and institutional policies that remove contextual barriers to produce habitual engagement in leisure physical activity. Fitness for all can lead to a nation that is more vibrant and inclusive. The economic, cultural, and social life of other nations can be enhanced immeasurably by the formation of a national fitness campaign that is tailored to national needs.

It should be acknowledged that, the study employed conventional questionnaire-based assessments, which may differ from the actual situation. Longitudinal experimental research on the effect of leisure physical activity on prosocial and antisocial behaviors is warranted to confirm our findings.

In conclusion, individuals who regularly participate in mass sports enjoy greater flow, which improves their subjective wellbeing and increases their prosocial behavior. Since the implementation of the National Fitness Program, China's public fitness system has been increasingly refined, and the number of individuals who regularly engage in mass sports activity has risen. The larger significance of this study provides empirical data to support the National Fitness Program's policy priority of increasing sports population in order to improve physical health and social cohesion. Moving forward, China's fitness for all that promotes a healthy society ought to be a global goal.

## Data availability statement

The data that support the findings of this study are openly available in figshare at doi: 10.6084/m9.figshare.20218715.v1.

## Ethics statement

The studies involving human participants were reviewed and approved by Hunan Normal University. Written informed consent to participate in this study was provided by the participants' legal guardian/next of kin.

## Author contributions

XD, XW, XL, SL, and YZ: conceptualization. XD and YZ: methodology. XD and SL: investigation. XD, XW, and SL: formal analysis. XD: writing—original draft preparation. XW, XL, SL, YZ, and TB: writing—review and editing. YZ: project administration. XW: funding acquisition. All authors have read and agreed to the published version of the manuscript.

## Funding

This research was funded by the Department of Education of Zhejiang Province: Research on the construction of the carrier for the co-education of civic and political science from the Sanquan Education perspective, Grant Number Y202147685.

## Conflict of interest

The authors declare that the research was conducted in the absence of any commercial or financial relationships that could be construed as a potential conflict of interest.

## Publisher's note

All claims expressed in this article are solely those of the authors and do not necessarily represent those of their affiliated organizations, or those of the publisher, the editors and the reviewers. Any product that may be evaluated in this article, or claim that may be made by its manufacturer, is not guaranteed or endorsed by the publisher.
